# Research toolbox and material design to meet the challenge of rechargeable Ca metal batteries

**DOI:** 10.1016/j.joule.2026.102401

**Published:** 2026-04-15

**Authors:** Olivera Lužanin, Tjaša Pavčnik, Deyana S. Tchitchekova, Alexandre Ponrouch, Jan Bitenc

**Affiliations:** 1 National Institute of Chemistry, Hajdrihova 19, 1000 Ljubljana, Slovenia; 2 Institut de Ciència de Materials de Barcelona, ICMAB-CSIC, Campus UAB, 08193 Bellaterra, Spain; 3 Alistore-European Research Institute, CNRS FR 3104, Hub de l’Energie, Rue Baudelocque, 80039 Amiens, France

**Keywords:** Ca rechargeable batteries, research methodology, metal anode, cathode materials, electrolyte, interphase

## Abstract

Ca metal anode batteries have emerged as a potential next-generation battery technology due to the low redox potential of Ca metal, high capacity, and abundance. However, practical development requires further improvements of electrolytes and cathode host materials. Recent advances in the design of weakly coordinating electrolytes have enabled reversible Ca plating/stripping and the testing of practical cathode materials.

However, the formation of blocking Ca metal interphases, insufficient electrolyte operating windows, and limited cathode reversibility pose fundamental challenges, requiring new strategies beyond current approaches—in particular, the development of a stable ion-conducting Ca metal interphase through the application of new Ca electrolytes and fine-tuning of ion solvation structures, as well as high-energy-density cathode hosts with improved reversibility. Currently, many literature studies are plagued by direct transfer of methodology from Li battery research, leading to insufficient characterization and erroneous conclusions due to peculiar Ca electrochemistry, which is impeding the progress of the field. Therefore, future Ca battery research should employ an improved research toolbox coupled with computational modeling to accelerate the exploration of chemical space.

## Context & scale

The introduction of Li-ion batteries (LIBs) has revolutionized our daily lives by enabling portable electronics and electromobility. However, the rapidly increasing demand for LIBs has led to a significant strain on the raw materials essential for Li-based energy storage systems and raised questions about their future price, availability, and, most importantly, the sustainability of their use. Calcium metal is an excellent candidate for next-generation batteries due to its low redox potential of −2.87 V (vs. standard hydrogen electrode, SHE) and high anode capacity. However, the development of Ca batteries is hampered by both a lack of practical electrolytes and cathode hosts. Significant progress has been made in recent years, particularly in the area of electrolytes that enable the reversible Ca metal plating/stripping and cathode materials that do not undergo traditional ion insertion mechanisms, such as organics and sulfur. Despite the progress, significant challenges still stand in the way of practical Ca battery application.

In this review, we aim to highlight the key obstacles that Ca metal batteries have to overcome on the way to practical viability. Improving Ca metal plating/stripping beyond 99% efficiency and developing highly reversible Ca^2+^ ion cathode hosts may require fundamentally different approaches than those currently in use. At the same time, computational modeling support can go a long way to improve our understanding of Ca electrochemistry and accelerate exploration of the available chemical space. When conducting studies on electrochemical Ca cells, researchers might be unaware of challenges connected with cell setups and electrolyte preparation that can affect experimental results. Therefore, complementary characterization techniques and comparisons across different cell setups are required to avoid erroneous conclusions and accelerate the development of Ca battery technology.

## Graphical abstract

**Figure:**
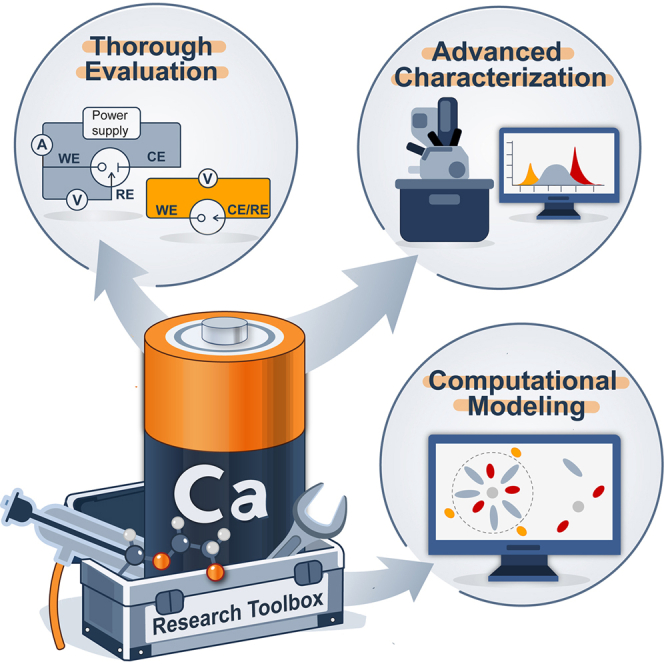

## Teaser

Ca metal anode batteries are considered potential next-generation high-energy-density batteries. However, their practical applications will require significant improvements in the design of the Ca metal interphase, electrolyte stability, and reversibility of cathode hosts. However, current literature reports are often based on insufficient research methodology directly imported from Li battery research, which frequently leads to erroneous conclusions and severely impedes material development. Therefore, an improved research toolbox is needed to accelerate the development of Ca metal batteries.

## Why Ca metal batteries

Among multivalent metals, Ca comes closest to Li in terms of its redox potential, with a difference of only about 0.15 V. At the same time, the Ca metal anode has a high gravimetric and volumetric capacity of 1,337 mAh/g and 2,073 mAh/cm^3^, respectively, offering a significant advantage over the state-of-the-art graphite anode (372 mAh/g, 837 mAh/cm^3^) used in current Li-ion battery (LIB) technology. The first Ca battery, the Ca-SOCl_2_ cell, was seen as an alternative to the Li-SOCl_2_ primary battery cell more than four decades ago. When the Ca primary battery was researched, the passivation of the Ca metal electrode was seen as a potential safety advantage over the Li counterpart, as it prevents the deposition of Ca and a possible short circuit of the cell.^1^ Unfortunately, the highly stable passivation layer also limited interest in the development of the rechargeable Ca battery. It was not until several decades later that the first electrolytes for reversible Ca metal plating were demonstrated.^2^^,^^3^ At the same time, the quest for more sustainable high-energy batteries has increased interest in Ca metal-based chemistry. However, the peculiarities of Ca metal, namely its low redox potential and associated metal passivation, currently limit both the reproducibility of experimental observations and the development of Ca batteries.

In Ca battery research, the evaluation of electrolytes and materials’ performance critically depends on the validity and limitations of the underlying experimental methods. Accordingly, this tutorial-style review is designed to provide researchers new to the field with foundational methodological guidance. It first establishes key experimental principles before discussing specific materials and reported performance claims. The novelty lies in combining a structured description of these principles with a critical evaluation of commonly used electrochemical methods, including a discussion of frequent measurement artifacts and stepwise best practices for assessing reported materials’ performance. Overall, the review aims to contextualize and, where necessary, reassess claims in the existing calcium-battery literature.

While the high gravimetric and volumetric energy densities of metal anodes make Ca metal one of the main candidates for next-generation high-energy batteries, the total energy density of the cell is determined by the combination of active cathode and anode materials through their respective potentials and capacities. Practical energy density modeling has shown that Ca batteries, with an already modest operating voltage, could potentially exceed the energy density of LIBs, assuming the use of similar electrode loadings and cell materials as in LIBs. A theoretical Ca cathode with a working potential of 3.5 V vs. Ca^2+^/Ca and a practical capacity of 250 mAh/g could lead to a practical cell energy density of 500 Wh/kg.^4^ Unfortunately, the electrochemical performance and reversibility of both the Ca metal anode and the various cathode materials remain far from practical, with the cell engineering parameters of Ca battery cells still unknown. Various classes of cathode electrode materials (insertion, conversion, and coordination) have been investigated over the last 10 years. While on the anode side, the pure Ca metal is clearly the preferred choice for future Ca batteries, Sn-based alloys have also been explored as alternative anode materials.^5^ However, before looking at specific parts of the Ca battery cell, let us first take a look at the specifics of Ca testing in electrochemical setups. While there are studies in the literature that deal with aqueous Ca batteries, these are not covered in this review. In our opinion, aqueous Ca batteries offer no distinct advantage over their similarly abundant alkaline analogs, such as Na and K, which have better overall cation mobility and should therefore offer better electrochemical performance.

### Toolbox for Ca electrochemical tests

#### Caveats of Ca electrochemical cell setups

**Figure 1:**
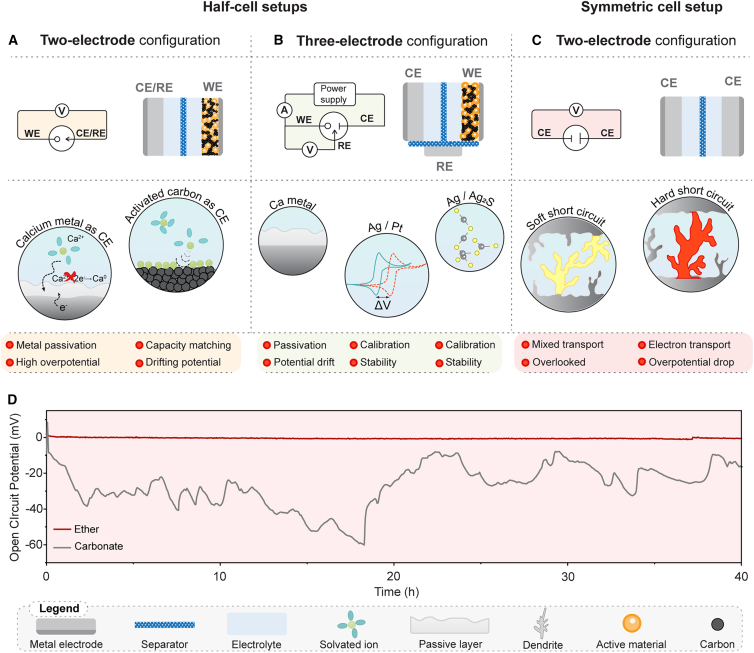
Overview of common Ca cell testing setups and their specific limitations (A and B) Half-cell setup with (A) 2 electrodes and (B) 3 electrodes. (C) Symmetric Ca metal cell setup. (D) OCP changes over the course of 40 h in Ca/Ca symmetric metal cells in Ca(B(hfip)_4_)_2_/G1 electrolyte and Ca(ClO_4_)_2_/ethylene carbonate:propylene carbonate (EC:PC) (1:1) (EC:PC data obtained from reference Tchitchekova et al.^9^).

A reliable electrochemical setup is crucial for evaluating any battery chemistry and even more so for less researched and peculiar systems such as Ca. In most common laboratory practice, the electrochemical performance of cells is tested using the so-called “half-cell” 2-electrode configuration that is a standard in Li battery research. Testing of Ca battery cells is usually done with two or three electrodes, where the cells are constructed with Ca metal as a counter electrode (CE) and/or reference electrode (RE). Unfortunately, CEs and REs made of Ca metal usually have very large overpotentials and limited reversibility and typically do not provide a reliable redox potential due to the inherent passivation of the Ca metal. Additionally, different impurities (e.g., oxygen, water, or solvent vapors) present in the electrolyte or glovebox during cell assembly can lead to the formation of blocking passivation films, making Ca metal electrode irreversible. Currently, reversible plating/stripping of Ca metal, in contrast to Li metal, has only been demonstrated in a few electrolyte formulations. Even in those electrolytes, relatively low Coulombic efficiencies and high overpotentials are observed on the Ca metal anode.^6^^,^^7^ Therefore, alternative CEs, such as activated carbon (AC) with capacitive storage (Figure 1A), have been adapted to enable testing of active material properties in different electrolyte formulations, allowing more flexibility in terms of used solvents and salts. However, the use of AC as CE should be done with great care regarding cell balance to avoid side reactions. For non-calciated materials, pre-calciation of the AC CE may be necessary to prevent the electrolyte dilution effect.^8^ As the AC CE potential is continuously adjusted to compensate for the capacity of the WE, the use of a stable RE is mandatory to allow control of the working electrode (WE) potential.

The RE should be simple to prepare and, above all, have stable and reproducible potential.^9^ It was found that Ca metal exhibits significant potential shifts and poor potential stability, which is highly dependent on the electrolyte used. Significant potential shifts have been reported in the literature, even in ether-based solvents, which are currently considered the most compatible with Ca metal anode.^10^ The extent of the potential shift is probably determined by the initial surface chemistry of the metal (e.g., the presence of oxides or hydroxides) and the composition of the electrolyte. Our measurements of the open circuit potentials (OCPs) of symmetric Ca/Ca metal anode cells have shown that the voltage fluctuations are much more pronounced in electrolytes that completely passivate Ca metal (Ca(ClO_4_)_2_/carbonate electrolyte) than in those that enable reversible plating/stripping (calcium tetrakis(hexafluoroisopropyloxy)borate salt [Ca(B(hfip)_4_)_2_]/dimethoxyethane (G1), Figure 1D). Other types of quasi-REs (QREs), such as AC, Ag, or Pt metal wires, have also been investigated.^7^^,^^8^ However, the exact identity of the “redox couple” that determines their potential is often not known, and calibration of the potential as well as stability assessment must be done using a known standard redox couple such as ferrocene (Fc) or cobaltocene (Cc), where the potentials of Fc^+^/Fc and Cc^+^/Cc are 0.4 and −0.92 V vs. SHE.^11^ To enhance the potential stability of RE, the surface chemistry of electrodes can be controlled through coatings (e.g., Ag/AgX, where X^−^ is ideally the anion present in the electrolyte).^8^^,^^12^

A very common approach in the current literature is the use of symmetric Ca metal cells for conducting plating and stripping tests, which helps in estimating the overpotential and lifetime of metal anodes. However, symmetric cell tests can often be misleading. For instance, the large overpotential and low reversibility of Ca plating/stripping can lead to electrolyte decomposition, which can be mistaken for reversible Ca plating/stripping. Secondly, plating/stripping tests in symmetric cells can favor short circuits. Short circuits can be either “hard” or “soft.” While hard short circuits are characterized by a sudden overpotential drop and a clear resistor-like cell response, soft short circuits can be difficult to observe, as the electrochemical response of the cell in a soft short circuit might not display an abrupt overpotential drop and often still resembles a “normal” cycling (Figure 1C). Therefore, cells with soft short circuits are often run for several hundreds of cycles, leading to the false conclusion of very stable metal plating/stripping behavior. Soft short circuits can be detected by electrochemical impedance spectroscopy (EIS) by determining the activation energy (Ea) at different temperatures or alternatively by using asymmetric cells. Such setups enable easier detection of short circuits because their cycling behavior is asymmetric but becomes symmetric in the case of hard shorts and exhibits potential instabilities in the case of soft shorts.^13^ Furthermore, Ca plating/stripping experiments should always be performed in parallel cells to check the reproducibility, as short circuits typically exhibit a certain degree of irregularity.

An additional layer of complexity in the repeatability of Ca cell measurements originates from the lack, as well as the quality, of commercial Ca foils. Typically, Ca metal electrodes are made by in-house pressing or punching of Ca metal shots or Ca dendritic pieces into metal discs, making the surface treatment, as well as foil thickness, unsystematic. Besides, since Ca metal foil is highly reactive, its quality and surface passivation can vary greatly between different sources and pretreatment procedures. It can also easily react with impurities inside the glove box atmosphere, such as oxygen, water, and solvent vapors, again resulting in potentially blocking passivation and a lack of reproducibility between different laboratories. Currently, most laboratories use mechanical scraping of the Ca foils just before assembling the cells, but here again, various scraping protocols can lead to unsystematic morphology and surface chemistry.

On the cathode side, several possible side reactions, such as electrolyte decomposition or corrosion, must be checked beforehand to ensure reliable testing. While the anodic stability of a given electrolyte can be roughly estimated using linear sweep voltammetry (LSV, necessarily performed in a 3-electrode cell configuration and at slow sweep rates) without the active material, LSV often leads to an overestimation of the electrolyte stability window. The latter is decreased in the presence of high-surface-area electrodes, especially in the presence of transition metals that can catalyze electrolyte decomposition reactions. Nevertheless, LSV provides initial qualitative information on the anodic stability of the electrolyte (or even more specifically on the anion stability if an anodically stable solvent such as acetonitrile [ACN] is used) and the corrosion resistance of current collectors.^8^^,^^14^ Yet, it is important to evaluate the electrochemical stability not only on flat metallic substrates but also on high-surface-area electrodes with cathode active material. It is worth mentioning that high corrosion resistance in Li-ion cells is often achieved by forming an AlF_3_-rich passivation layer in a PF_6_^−^ based electrolyte. However, Ca^2+^ migration through such passive layer could be excessively slow and limit reversible electrochemical activity. On the anode side of the cell, there is still a lack of information on the possible formation of alloys (with Ca) and the effects of different substrates on the deposition kinetics and adhesion of the Ca metal deposits.

#### Ensuring Ca electrolyte quality

In our recent study, three different grades of G1 solvent were investigated in Ca electrolytes, and all solvent grades underwent an extensive three-stage solvent drying/purification process before use.^14^ Remarkably, only the electrolyte prepared from the solvent with the highest initial quality allowed reversible Ca metal plating/stripping. Electrolytes with moderate-quality solvents exhibited a significant deterioration in electrochemical performance, attributed to the passivation of the Ca metal by various impurities, indicating the high sensitivity of Ca metal. Therefore, researchers should always strive to use solvents of the highest possible quality to enable good reproducibility. This often translates into performing additional purification steps. The most common purifications in the laboratory are aimed at removing water with molecular sieves. However, other impurities, such as solvent inhibitors commonly present in ether solvents, can also lead to a detrimental passivation of the Ca metal anode.^15^^,^^16^ To tackle this issue, solvents are usually purified by distillation after prolonged reactions with various scavengers such as Na/K alloy, Na metal, CaH_2_, activated alumina, etc. The scavenging reagents differ in their effectiveness and solvent compatibility as well as the safety precautions required during their use. It is important to note that cleaning procedures should always be carried out by well-trained personnel utilizing prescribed personal protective equipment (PPE), especially when handling extremely pyrophoric material, e.g., alkali metals or Na/K alloy.

The limited availability of commercial Ca salts brings additional challenges to the preparation of Ca electrolytes. Moreover, conventional electrolytes using commercial salts usually show poor performance due to either the reductive decomposition of the salt anions (TFSI^−^ [bis(trifluoromethanesulfonyl)imide anion], ClO_4_^−^) or their limited oxidative stability (BH_4_^−^). The synthesis of new salts belonging to the class of weakly coordinated anions (WCAs) salts (carboranes and fluorinated alkoxyborates/aluminates) has improved the performance of electrolytes, particularly with respect to the cathode material compatibility and electrochemical stability window. However, the synthesis of these salts usually requires the use of highly reactive reagents, which necessitates careful handling by experienced personnel under controlled conditions and subsequent purification.^17^ In addition, some of the reagents are expensive or available only with moderate purity. The synthesis process may also require the use of specialized equipment (gloveboxes equipped for chemical synthesis, Schlenk lines…) that is not always available in typical electrochemical laboratories and may require collaboration with laboratories specializing in organometallic chemistry.

An alternative to the purification of solvents and salts is the conditioning of Ca electrolytes, which can be carried out chemically or electrochemically. Chemical conditioning usually involves mixing with metals or other reductive species, similar to the removal of impurities in solvents, while electrochemical conditioning is based on some form of electrochemical pre-cycling (galvanostatic cycling, voltammetry), where metal deposits are typically formed and reacted *in situ* with electrolyte impurities.^18^^,^^19^ In addition to changing the electrolyte composition, electrochemical conditioning also alters the electrode/electrolyte interphase when performed in the same cell as the subsequent electrochemical characterization. Although the conditioning procedures are effective, experimentally less complicated, and performed directly on the Ca electrolyte, they offer less control and can be very difficult to reproduce in different laboratories using different cell configurations.

#### Good practice for investigating Ca electrochemistry

**Figure 2:**
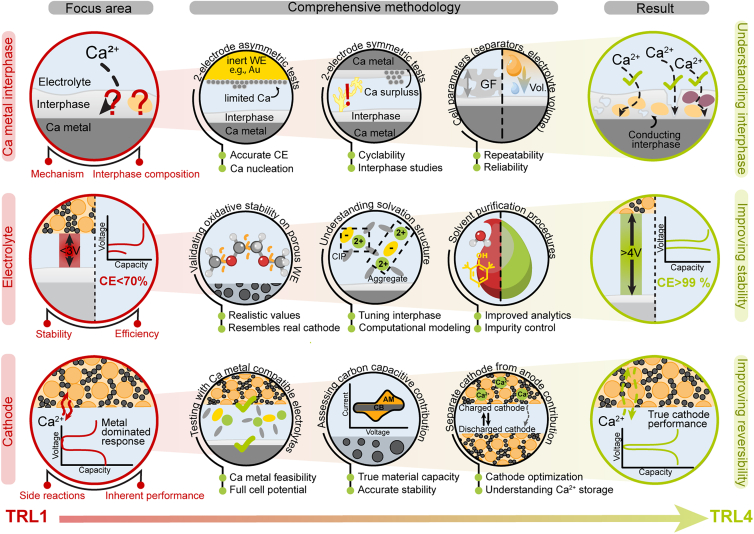
Overview of three key areas of Ca battery research (Ca metal interphase, electrolyte, and cathode materials) and methodology recommendations to improve the reproducibility and reliability of the obtained results GF and CE abbreviations denote glassy fiber separator and Coulombic efficiency, respectively.

In the previous two sections, we exposed several issues encountered in Ca electrochemical testing, but unfortunately, there is no simple silver bullet cell setup or electrolyte preparation procedure to ensure fully reliable experimental results. Instead, researchers need to be aware of the peculiar character of Ca electrochemistry and use different complementary materials and electrochemical characterization approaches to verify the obtained results (Figure 2).

As discussed previously, one of the most common mistakes is claiming reversible Ca plating/stripping from results of symmetric Ca metal plating/stripping tests, which often originate from side reaction contributions and soft short circuits. Therefore, we suggest complementary characterization in half-cell setups with inert WEs (such as Au or Pt) and with electrode materials that have already demonstrated reversible electrochemical storage of Ca^2+^ ions. It must be noted that even half-cell setups can be prone to issues with soft short circuits or side reaction contributions, which influence their electrochemical response. While both can significantly reduce reversibility of cells, contributions of side reactions should also lead to significantly modified cell potentials. At the same time, reversible Ca plating/stripping should be unambiguously confirmed with complementary material characterization tools such as X-ray diffraction (XRD) and electron microscopy. Use of 3-electrode cell setups with RE can help distinguish between electrode contributions and help to identify key overpotential contributions. However, given the lack of a reliable RE, its potential should always be carefully checked in a specific electrochemical system. At the same time, cell parameters such as separator, current collectors, and electrolyte volume should also be monitored and consistently reported to increase both result reliability and reproducibility across different laboratories.

Ca electrolyte quality and solvation structure can have a big impact on the electrochemical performance of Ca cells. Therefore, researchers should carefully characterize their electrolytes to better understand the electrolyte stability window, solvation structure, and role of impurities. For example, past results have already shown a positive effect of Na^+^ cation impurities^20^ and a negative effect of glyme solvent impurities.^14^ Thus, future research should take a more systematic approach to studying the effect of impurities and introduce additional analytical techniques, such as gas and liquid chromatography to determine organic impurities and elemental analysis techniques to identify metal impurities. While this will inherently require significantly more effort than current approaches, it is essential for improving our understanding of Ca electrochemistry, increasing the reliability of experimental results, and helping explain discrepancies between different experimental reports.

On the cathode side, many research reports use cathode structures that contain significant amounts of water^21^ or even water as a co-solvent in the electrolyte,^22^ which makes Ca metal anode compatibility highly unlikely. Many literature reports also use large amounts of conductive additives in electrode formulation. This often leads to considerable overestimation of material capacity due to additional capacitance contributions, which are typically not accounted for.^23^ Complementary testing of cathode materials in setups that eliminate overpotential contributions from Ca metal (such as 3-electrode setups, symmetric cells, or AC CE) should be performed to obtain the inherent electrochemical performance of the cathode and improve understanding of material kinetics.

### Ca electrolyte development

#### Path toward first Ca electrolytes enabling reversible metal plating/stripping

**Figure 3:**
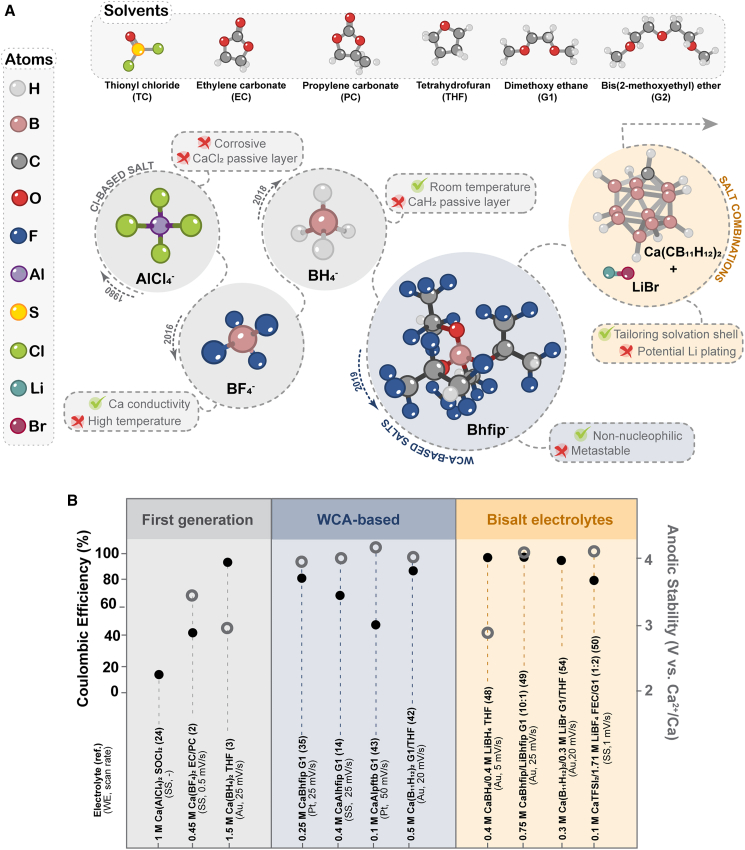
Schematic evolution of Ca electrolytes for reversible Ca metal plating/stripping (A) Different generations of Ca electrolytes with specific strengths and limitations. (B) Coulombic efficiency of metal plating/stripping and anodic stability (where available) for key representatives of different Ca electrolyte generations. SS abbreviation denotes stainless steel electrode.

The first practically tested Ca electrolytes were analogs of Li electrolytes from primary Li-SOCl_2_ batteries. Initially, only Ca metal stripping was observed in Ca(AlCl_4_)_2_/SOCl_2_.^1^ However, later, Ca plating was also observed, but with very low Coulombic efficiency and extremely high overpotentials (Figure 3).^24^ The main reason for this was the blocking nature of the CaCl_2_-based passive layer. A comprehensive study tested the behavior of Ca metal electrodes in various solvents (ACN, tetrahydrofuran [THF], propylene carbonate [PC], and γ-butyrolactone [GBL]) and Ca salts, similar to the Li electrolytes. No Ca deposition could be observed in any of the electrolytes tested, and Ca stripping only occurred at very high overpotentials.^25^ The first breakthrough was the reversible Ca plating/stripping in Ca(BF_4_)_2_ in a carbonate mixture EC:PC=1:1 at elevated temperatures. In this study, it was shown that the electrochemical behavior of plating/stripping is influenced by the concentration of the salt, with the need to find a compromise between ionic conductivity and limited ion pairing.^2^ The Ca metal interphase in Ca(BF_4_)_2_ and Ca(TFSI)_2_/EC:PC electrolytes was afterward investigated by X-ray photoelectron spectroscopy (XPS), revealing significant differences between the salts used.^26^ With Ca(TFSI)_2_/EC:PC salt, a relatively thin interphase consisting mainly of carbonates and only a small amount of CaF_2_ (2%) could be detected. Using Ca(BF_4_)_2_ salt, a thicker, yet Ca-ion-conducting, interphase was observed, rich in organic and boron species. The low amount of TFSI^−^ anion decomposition products was attributed to the low contact ion pair (CIP) concentration in this electrolyte, as Ca^2+^ cations are mostly coordinated by the solvent and produce mainly solvent-derived decomposition products. Later, reversible Ca plating/stripping was achieved using Ca(TFSI)_2_/EC:PC-based electrolyte with boron trifluoride, BF_3_ additive (stabilized in diethyl ether, i.e., BF_3_·Et_2_O).^27^ Later, different BF_3_ solvent adducts were successfully prepared and used as additives for the formation of Ca borate-rich interphase similar to the one obtained in Ca(BF_4_)_2_/EC:PC electrolyte, allowing reversible Ca plating/stripping in Ca(TFSI)_2_ electrolyte.^27^^,^^28^

The development of ether-based Ca electrolytes was motivated by the successful development of Mg electrolytes and the greater reductive stability of ether solvents compared with carbonates. The first electrolyte to enable Ca metal plating/stripping at room temperature (RT) was Ca(BH_4_)_2_/THF.^3^ In contrast to most previously tested electrolytes, where CaCO_3_, Ca(OH)_2_, and Ca alkoxides were formed on the surface of Ca metal, CaH_2_ was initially reported on the surface of Ca deposits, which did not hinder the Ca plating/stripping, and Coulombic efficiency up to 96% was observed. Nevertheless, Ca metal spontaneously reacted with the electrolyte during cell rest, resulting in a continuous growth of CaH_2_ layer and decreased plating/stripping efficiency. This implied that CaH_2_ does not behave as an ideal “Li-like” SEI (solid electrolyte interphase). The initial analysis of Ca(BH_4_)_2_/THF metal/electrolyte interphase was later challenged, as CaH_2_ was observed as inclusions within the Ca metal deposit, and the surface was found to be covered by a heterogeneous interphase consisting mainly of CaO, with minor amounts of Ca borate and CaCO_3_.^20^ Another work calculated the migration energy barriers of Ca^2+^ cations in different potential Ca-based SEI compounds by density functional theory (DFT). Although very high barriers were found for CaCO_3_ (1,438 meV), it was surprising that the barrier for CaO is significantly lower (997 meV).^26^ While a 700 meV barrier is commonly considered too high to cycle electrode active material at an acceptable rate for a practical battery, the value for an acceptable barrier strongly depends on the operating temperature and layer thickness. Therefore, reasonable rate performance could be expected for nanoscale interphase, such as the native CaO surface oxide. This is in stark contrast to MgO, with a prohibitive migration energy barrier of 1,856 meV for Mg^2+^ ion. It is worth noting that CaH_2_ has the lowest migration energy barrier for Ca^2+^ diffusion among all investigated candidates.^26^

The solvation structure of Ca(BH_4_)_2_/THF was intensively characterized by a combination of experimental and computational modeling techniques.^29^^,^^30^ It was revealed that a variety of Ca species form in this electrolyte, including neutral monomers, dimers, and ionic clusters. Their population evolves with salt concentration, with CaBH_4_^+^ presumably being the electroactive cationic species responsible for the release of Ca^2+^ to the electrode surface for metal deposition. Although the CaBH_4_^+^ species population was found to slightly increase with the salt concentration, its relative population was quantified at less than 0.1 M, even above 1 M salt concentration, highlighting the multitude of Ca^2+^-coordination structures in THF. Separate computational screening through a combination of DFT and conductor-like screening for real solvent approach (COSMO-RS) revealed that BH_4_^−^ anions tend to bind Ca^2+^ cations very strongly in most conventional solvents and that the dissociation energy of the ion pairs formed is higher than for other salt anions typically used in LIB electrolytes (BF_4_^−^, PF_6_^−^, and TFSI^−^).^31^ The molecular dynamics (MD) investigation of electron transfer between Ca metal and different solvent molecules showed that tetraglyme (G4) is stable, while EC and PC both decomposed on the Ca metal surface.^32^ The calculated barriers associated with the cleavage of different bonds in EC and PC molecules on the Ca metal are comparable to the energy fluctuations at RT (20–70 meV), which is consistent with the experimental evidence for the decomposition of carbonates on the surface of Ca metal. However, solvent molecules (and anions) coordinating Ca cations are significantly more prone to being reduced on the surface of the Ca metal due to strong interactions, and glyme solvent reduction during Ca electrodeposition cannot be excluded.^33^

#### New generation of electrolytes based on weakly coordinating anion salts

A key step forward was achieved by the introduction of Ca electrolytes based on WCA salts. This approach was demonstrated concurrently by two groups who synthesized Ca(B(hfip)_4_)_2_, which showed reversible Ca plating/stripping and oxidative stability beyond 4 V vs. Ca^2+^/Ca in G1.^34^^,^^35^ Recently, a more cost-effective general synthesis route toward different Ca alkoxyborate salts has been developed through cation replacement from Zn(BH_4_)_2_ precursor.^36^ Favorable electrochemical properties of Ca(B(hfip)_4_)_2_/G1 together with its non-nucleophilic character paved the way to investigate cathode materials with higher operating potential and electrophilic character (sulfur and organics) in metal anode cells. The main compound present at the Ca metal interphase in Ca(B(hfip)_4_)_2_-derived solutions was initially identified as CaF_2_, the latter most likely not forming a conformal layer, as it could block cation transport and reversible plating, given the calculated 2 eV diffusion barrier for Ca^2+^ in CaF_2_.^26^ MD modeling of the anion stability vs. Ca metal slab showed that breakage of a limited number of C–F bonds can occur, corroborating the formation of CaF_2_ on the Ca surface.^32^

The electrochemical performance of Ca(B(hfip)_4_)_2_ was later tested in different ether-type solvents (THF, G1, and diglyme [G2]) and in combination with different current collector substrates (glassy carbon [GC], Pt, Cu, and Al).^37^ The highest Ca plating/stripping efficiency was achieved in G2 on GC WE. Remarkably, the largest amount of fluorine was found during reduction in Ca(B(hfip)_4_)_2_/THF, compared with G1 and G2 solutions, where mainly oxygen and carbon were found on the Ca deposits and, to a smaller extent, fluorine. These differences in the elemental composition of electrodeposits can be rationalized by considering the coordination ability of the various solvents. THF being a weakly binding solvent, the resulting Ca^2+^ solvation shells are more flexible and may allow stronger interactions with nearby anions, facilitating the decomposition of B(hfip)_4_^−^. On the other hand, glyme chains can coordinate Ca^2+^ in bidentate, tridentate, or even tetradentate configurations, depending on chain length and conformation. Longer glymes enable higher denticity and more stable chelating solvation structures, resulting in comparatively more rigid Ca^2+^ solvation shells.^33^ Thus, mainly solvent molecules are decomposed in the interphase. A recent study has shown that the pre-passivation of Ca metal in Ca(B(hfip)_4_)_2_/G1 electrolyte has a positive effect on the reversibility of Ca metal anode, while the electrochemical cycling of untreated Ca metal displayed significantly higher interfacial resistance.^38^

The interplay between solvent- or anion-derived decomposition during electrochemical cycling in the same electrolytes was found to be concentration dependent, with actual Ca(B(hfip)_4_)_2_ dissociation being lower in dilute glyme solutions of 0.01 M, where solvent-separated ion pairs were predominantly present.^39^ Later, another study investigating the effect of glyme chain length showed an opposite trend of decreasing plating/stripping Coulombic efficiency from G1 to G2 and triglyme (G3), which was attributed to more difficult desolvation with longer glymes.^40^ However, it may be difficult to separate the effect of solvent purity from the effect of solvent coordination, as longer glymes are available with lower purities and are much more difficult to purify.^15^ Limited reversibility of Ca deposition due to high desolvation energy when Ca(B(hfip)_4_)_2_ is dissolved in longer chain glymes was also corroborated by a potential-dependent DFT study.^41^ It was found that electron transfer from a Ca metal surface to the fully coordinated complex [Ca(G4)_2_]^2+^ (coordination number [CN] of 8) is not possible. Rather, “solvated electrons” would form in the second solvation shell of this species and thus prevent the efficient reduction of the Ca^2+^ center. By examining the free electrochemical energy of other plausible species, the authors observed that the fully coordinated [Ca(G4)_2_]^2+^ complex could partially desolvate to an electroactive-[Ca(G4)_2_]^2+^ complex of CN 6, with nearly an 800 mV overpotential penalty, which supports the sluggish kinetics for Ca electroplating in G4. Directions to stabilize partially desolvated Ca^2+^ species, such as the development of solvate ionic liquids or the use of weakly coordinating co-solvents, are being discussed as guidance for future Ca electrolyte development.^41^

The application of boron-based WCA salts in Ca electrolytes was further explored with the synthesis of Ca monocarborane salt (Ca(CB_11_H_12_)_2_) in a THF/G1 mixture, which exhibited a Ca plating/stripping efficiency of up to 88% and high oxidative stability.^42^ Due to the absence of fluorine in the anion structure, no CaF_2_ was formed at the Ca metal/interface. However, considerable amounts of O and C and small amounts of B were detected on the surface of the Ca metal deposits by EDS (energy-dispersive X-ray spectroscopy), indicating the decomposition of the solvent and, to a lesser extent, the anion. Besides metallic Ca, the presence of CaH_2_ was detected in the XRD diffractograms of the Ca metal electrodeposits. A DFT study of [Ca(G1)_3_]^2+^ complexes found a positive change in free energy when one solvent molecule was exchanged by one CB_11_H_12_^−^ anion, thus suggesting a thermodynamically unfavorable coordination of Ca^2+^ by this anion, supporting their classification as WCAs.^43^ An MD study of the CB_11_H_12_^−^ anion stability in contact with a Ca metal surface did not observe any bond breaking, even at elevated temperatures, indicating good stability of the monocarborane anion on Ca metal.^32^

The concept of boron-based WCA salts has also been extended to the class of aluminates. Two fluroalkoxyaluminate salts have demonstrated reversible Ca deposition: Ca(Al(hfip)_4_)_2_, an aluminate analog of Ca(B(hfip)_4_)_2_,^14^^,^^44^ and calcium tetrakisperfluoroterbutoxyaluminate (Ca(Al(pftb)_4_)_2_),^43^ which is based on a perfluorinated alkoxy ligand without potentially labile protons. Aluminate-based electrolytes exhibited moderate Coulombic efficiency in Ca plating/stripping (up to 70% and 55% for Ca(Al(hfip)_4_)_2_ and Ca(Al(pftb)_4_)_2_, respectively), which was lower than previously published results for fluoroalkoxyborate salts. Unfortunately, a direct comparison of the literature reports from different groups is not possible due to the different cell setups, WEs, electrolyte concentration, and the presence of impurities. In a direct benchmarking of Ca(Al(hfip)_4_)_2_ and Ca(B(hfip)_4_)_2_ electrolytes performed by our group, Ca(Al(hfip)_4_)_2_ showed marginally better Coulombic efficiency (70% vs. 65% for steady-state cycling), as well as lower overpotential in Ca metal-organic full cells.^14^ EDS characterization of the Ca metal deposits in both electrolytes showed large amounts of C and O elements and relatively low amounts of F. This is in sharp contrast to CaF_2_, which was initially reported as the main component of the Ca metal/electrolyte interphase in the Ca(B(hfip)_4_)_2_. *Ex situ* EDS characterization of Ca metal electrode in contact with the solvent and electrolyte revealed that decomposition of G1 solvent occurs primarily and appears to be more intensive on the electrochemically deposited Ca than on pristine bulk Ca metal.^14^ Although ether solvents have better reductive stability than carbonates, current electrolyte formulations do not enable the formation of a stable interphase with Ca metal, as continuous interfacial impedance growth is commonly observed upon cycling.^45^ At the moment, it seems that only metastable behavior can be achieved with the current generation of Ca electrolytes. A computational comparison of the kinetics of reductive anion decomposition of different aluminates and borates has shown that B–O bond breaking in borate-based salts seems to be mainly determined by steric effects of the ligands, whereas in the case of aluminate-based salts, electron-withdrawing effects seem to be more important.^46^ Recently, the surface-specific reactivity of fluorinated Ca-aluminate and borate salts toward a (001) Ca metal plane was investigated by means of DFT and *ab initio* MD (AIMD) simulations.^47^ CaF_2_ was confirmed as the most prominent anion decomposition product, but carbide-like motifs were also found to form on the Ca surface. At the same time, it was predicted that sterically less hindered anions with a moderate degree of fluorination, such as the trifluoroisopropanol (tfip)-based anions, in contrast to the hexafluoroisopropanol (hfip)-containing analogs, do not undergo reduction near the Ca surface, making them promising candidates for future salt synthesis. In general, the aluminate salts were found to be less reactive with the Ca surface than their borate analogs, which is a strong argument for their further exploration. Overall, there is a clear need for a combined synthetic and computational approach to find the optimal anion structure for the next generation of WCA-based Ca salts.

#### Beyond weakly coordinating anion Ca salts

In recent years, novel electrolyte design strategies have been proposed to modify the Ca metal interphase and, consequently, improve electrochemical performance by tailoring the first solvation shell of Ca^2+^ ions. One of the commonly used approaches is the addition of Li salts to Ca-based electrolytes. To date, the highest reported efficiency of metal plating/stripping of over 99% has been achieved in a dual salt electrolyte of Ca(BH_4_)_2_ and LiBH_4_ in THF solvent. The success of this electrolyte was attributed to the modification of the Ca^2+^ solvation shell by Li^+^ cations, which decreased the CN of Ca^2+^ ions.^48^ More recently, a similar dual-cation strategy has been applied to WCA-based electrolytes. In the Ca[B(hfip)_4_]_2_/G1 solution, the addition of Li[B(hfip)_4_] promoted the formation of mixed Ca^2+^-DME and Ca^2+^-Li^+^-B(hfip)^−^ species, which led to the formation of an SEI composed of a flexible organic matrix and borate-rich inorganic compounds, with a significantly reduced CaF_2_ content compared with the pristine Ca[B(hfip)_4_]_2_ electrolyte. As a result, the Coulombic efficiency increased from 48% to 97% upon the addition of Li[B(hfip)_4_].^49^ The combination of Ca(TFSI)_2_ with LiBF_4_ has also been explored to obtain an anion-derived inorganic-rich metal interphase.^50^ The dual-cation (Ca^2+^/Li⁺) concept has also been extended to gel polymer electrolytes.^51^ Cyclic voltammetry and galvanostatic measurements of a Ca(CB_11_H_12_)_2_/LiBH_4_-based electrolyte within a polyTHF solution matrix demonstrated reversible metal plating/stripping, with improved oxidative stability and high stability against Ca metal anodes. Although the addition of Li salts clearly shows a positive effect on electrochemical performance, the question of potential Li plating and its contribution remains largely unanswered. This aspect is of particular importance considering the high Li salt concentration used in some of these experiments as well as the very small difference in standard potential between Ca and Li.

Another route toward improved metal/electrolyte interphase stability was pursued through the addition of bromide and iodide salts, which promote the formation of interphase layers containing halogen species^52^ and decrease the coordination of Ca^2+^ species with glyme solvents,^53^^,^^54^ both of which have been shown to have a positive effect on the electrochemical performance. For instance, a Ca(CB_11_H_12_)_2_/LiBr electrolyte in G1/THF exhibited a higher Coulombic efficiency and lower overpotentials compared with the pristine Ca(CB_11_H_12_)_2_ electrolyte.^54^ XPS analysis shows that the pristine electrolyte forms a CaCO_3_-rich SEI, mainly due to solvent reduction, whereas LiBr addition leads to an SEI composed of organic boron compounds and Br-rich species arising from anion-involved reduction processes. Similar behavior was observed upon the addition of CaBr_2_ to a Ca(CB_11_H_12_)_2_ electrolyte in G2.^53^ In addition, combining Ca(BH_4_)_2_ with Ca(CB_11_H_12_)_2_ has been shown to reduce the plating/stripping overpotential through utilization of solvent-separated ion pairs, Ca(CB_11_H_1__2_)^+^, instead of CIPs, Ca(BH_4_)^+^, as the main Ca electroactive species.^55^ Solvation shell modification was also explored through the use of multifunctional ligands that contained both ether and amine functional groups, which significantly improved Ca metal plating/stripping efficiency.^56^ However, unlike in the field of Mg electrolytes, the use of solvation sheath additives such as alkyl phosphates and related compounds remains largely unexplored for Ca electrolytes,^57^^,^^58^ and there is a lack of follow-up publications on the use of these additives.

An alternative approach is the use of solvents with higher donor numbers in combination with commercial Ca salts, resulting in a solvent-dominated cation solvation shell, such as Ca(TFSI)_2_/dimethylacetamide (DMAc)^59^ and Ca(OTf)_2_ in mixed N-methylacetamide/trimethylphosphate (OTf = trifluoromethanesulfonate).^60^ The reversible Ca plating/stripping in these electrolytes is attributed to an organic-rich SEI, rather than a more inorganic passive layer that typically forms in weakly solvating solvents. It should be noted that limited direct evidence for reversible Ca plating/stripping was provided exclusively in symmetric Ca/Ca cell setups without plating on an inert electrode, such as Au, and subsequent confirmation of Ca metal deposits by XRD. Recently, the Ca(AlCl_4_)_2_/SOCl_2_ electrolyte has been revisited with a Li difluoro(oxalate)borate salt (LiDFOB) as a mediator.^61^ While 90% Ca plating/stripping efficiency was claimed, very limited electrochemical characterization of the Ca metal anode was presented.

Moving beyond conventional liquid electrolytes, ionic liquids have attracted attention due to their low volatility and high thermal stability, offering improved safety for practical applications. Notably, Ca(BH_4_)_2_ dissolved in an alkoxy-ammonium-based ionic liquid, [N_07_][TFSI] (N,N,N-tri-(2-(2-methoxyethoxy)ethyl)-N-(2-methoxyethyl)ammonium bis(trifluoromethylsulfonyl)imide),^62^ enabled Ca plating/stripping. The favorable electrochemical performance was attributed to a displacement of TFSI^−^ from the Ca^2+^ coordination sphere by the alkoxy-functionalized ammonium cation. More recently, Ca plating/stripping was started with Ca(TFSI)_2_ salt in a mixture of the ionic liquid EMIMBF_4_ (EMIM-1-ethyl-3-methylimidazolium cation-EMIM^+^) and dimethyl sulfoxide (DMSO).^63^ Although reversible Ca plating/stripping was claimed, even in the presence of O_2_, very vague electrochemical characterization of Ca plating/stripping was presented, and Coulombic efficiency was not even reported.

Overall, recent studies demonstrate that engineering the Ca^2+^ solvation shell is key to controlling Ca metal interphase formation and improving the reversibility of Ca plating/stripping. WCA-based Ca electrolytes marked a major breakthrough by providing high oxidative stability and reversible Ca deposition, but they suffer from insufficient Coulombic efficiency and unstable metal/electrolyte interphases. Although a passivation-free Ca metal surface remains unlikely, Ca electrochemical reversibility should be governed by the nature of the interphase formed through controlled electrolyte decomposition. Interphase components predominantly originate from species present in the primary Ca^2+^ solvation shell, including coordinated solvents and anions. Across different electrolyte concepts, interphases rich in organic boron-containing species and halogen-derived components consistently correlate with improved Coulombic efficiency, reduced overpotentials, and enhanced cycling stability, indicating a promising direction for electrolyte design. Ca electrolytes also offer unique opportunities for interphase engineering, as divalent Ca^2+^-anion complexes retain a net positive charge and can migrate toward the electrode interface, unlike neutral ion pairs in monovalent systems, thereby expanding the range of electrolyte species that can be deliberately reduced to form favorable interphases.

### Cathode materials

Compared with the strides forward made in the field of Ca electrolytes, the progress in cathode active materials for Ca batteries has been comparatively stagnant, with more incremental improvements over the last 10 years. This is especially true for inorganic insertion-type materials, where the cathode performances are affected by the limited reversibility of the electrochemical redox process and the sluggish diffusion generally associated with divalent cations.^64^ Although the size of the Ca^2+^ cation is similar to the size of the Na^+^ ion and its charge density is much lower than in the case of the Mg^2+^ cation, Ca^2+^ has a lower number of hosts than Mg^2+^. Reversible Ca^2+^ insertion/deinsertion seems to face challenges arising from a combination of size and bivalent charge, as well as crystal chemistry considerations such as preferred cation sites.^6^ The higher charge density is particularly impactful on the solid-state diffusion kinetics due to stronger Coulombic interactions with the anion sublattice. Limited electrochemical reversibility can also be partially attributed to the poor stability of Ca electrolytes, which often leads to electrolyte decomposition instead of Ca^2+^ insertion/deinsertion, especially in the case of non-aqueous electrolytes.

#### Traditional insertion-type cathode materials

Insertion-type materials, where the redox mechanism involves the reversible topotactic insertion of ions into a crystal host bearing interconnected sites into which the guest ions can diffuse, were largely studied as Ca cathodes, motivated by their successful use in LIBs. The different classes of inorganic insertion materials that have been explored with Ca^2+^ so far can be classified as oxides, sulfides, polyanions, and metal-organic frameworks such as Prussian blue analogs (PBAs). In our review, we will not provide an in-depth overview of all inorganic materials explored but refer readers to other more focused reviews.^6^^,^^65^^,^^66^

Transition metal sulfides such as TiS_2_, traditionally known for their rich intercalation chemistry with various cations, were explored as potential Ca hosts and have shown reversible Ca insertion with high-voltage hysteresis, although accompanied by solvent co-insertion in various carbonate-based electrolytes at very low rates.^67^^,^^68^ A certain improvement was obtained using VS_4_ clusters nanostructured with rGO (reduced graphene oxide), which allowed electrochemical reversibility at RT in combination with a Ca metal anode and Ca(B(hfip)_4_)_2_ electrolyte.^69^ A mixed cation-anion redox activity (redox state of both V and S changed) was found to be responsible for VS_4_ electrochemical activity.

Among oxides, a family of compounds that have been intensively studied is layered vanadium oxide compounds, due to the promise of the high redox potential of V^+5^/V^+4^ redox couple. Vanadium oxides have been investigated in both the hydrated^70^ and water-free forms of different V_2_O_5_ polymorphs.^71^ However, the latter study concluded on the unsuitability of V_2_O_5_ as a Ca cathode in dry conditions and cautioned on misinterpretation of H^+^ for Ca^2+^ insertion. Layered α-MoO_3_ is another widely explored oxide, although reversibility of the redox process in the 1^st^ cycle and capacity retention were found to be quite low.^72^ Recently, it was shown that significantly improved electrochemical performance can be obtained by nanosizing MoO_3_ particles.^73^ Additionally, DFT-based and experimental studies investigated alternative Ca-insertion chemistries with ternary oxides. The CaMn_2_O_4_ spinel^74^ and the Ca_3_Co_2_O_6_ 1D framework^75^^,^^76^ presented limited reversibility for Ca insertion. DFT screening procedures have identified a few other promising oxide frameworks: the oxygen-deficient Fe-based oxides in the brownmillerite structure,^77^ with the Ca_4_Fe_9_O_17_ having the lowest cation migration energy barrier,^78^ the post-spinel-CaV_2_O_4_, and the layered-CaNb_2_O_4_.^79^ Although some experiments indicate promise on reversible Ca (de)insertion in different ternary oxides, proof of Ca deinsertion is presently difficult to obtain due to their high operational voltages, beyond the electrochemical stability of current Ca electrolytes.^78^^,^^80^

PBA materials, hexacyanometallate compounds with open and tunable structures and large channels, should facilitate Ca^2+^ ion diffusion. Another advantage of PBAs is their facile synthesis at RT by co-precipitation reactions, making them cheap and affordable for large-scale applications. So far, different types of PBAs have been explored as Ca cathodes, employing transition metals such as Mn, Fe, and Ni, all displaying reversibility in non-aqueous Ca electrolytes, but unfortunately, water content in these systems was not monitored, making H^+^ insertion contributions in electrochemical response quite likely.^81^^,^^82^

**Figure 4:**
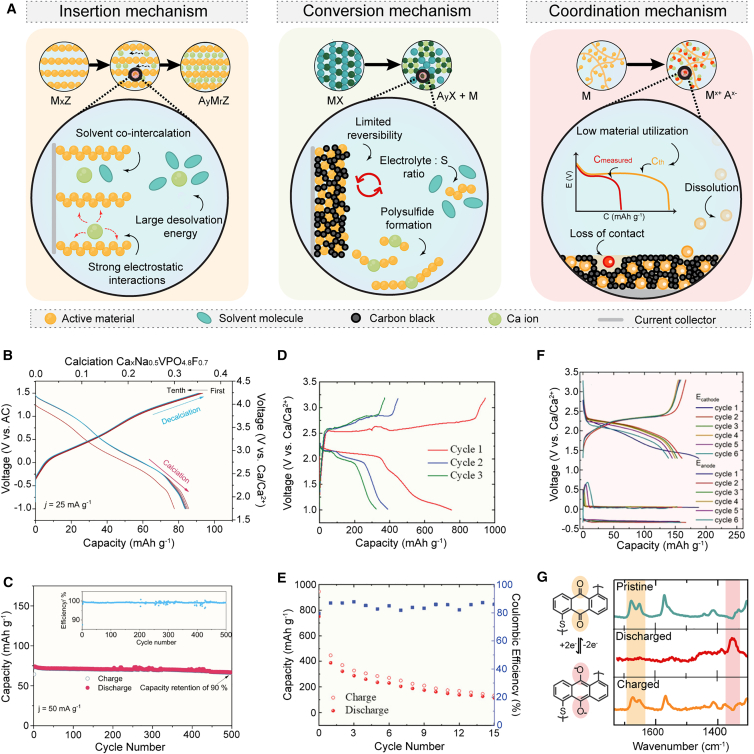
Overview of three different cathode electrochemical mechanisms that are typical in Ca batteries (A) Illustration of the issues characteristic of insertion cathodes, sulfur as an example of a conversion cathode, and organic electrodes. (B and C) Galvanostatic curves and capacity retention over 500 cycles of Na_1.5_VPO_4.8_F_0.7_ cathode host in Ca(PF_6_)_2_ EC/PC electrolyte. Adapted from reference Xu et al.^87^ under CC BY 4.0. (D and E) Galvanostatic curves and capacity retention of sulfur cathode in Ca(B(hfip)_4_)_2_ electrolyte. Adapted from reference Li et al.^92^ under CC BY 4.0. (F and G) Galvanostatic curves of the PAQS electrode in Ca(B(hfip)_4_)_2_ and *ex situ* IR spectroscopy with characteristic carbonyl and enolate peaks labeled in orange and red, respectively. Adapted from reference Bitenc et al.^45^ under CC BY 4.0.

Another interesting class of inorganic compounds are polyanions compounds, which, in the form of LFP (lithium iron phosphate) cathode material, have taken over the market of LIBs. Among polyanion compounds, several different types were investigated, ranging from FePO_4_,^37^^,^^83^ Na_2_FePO_4_F,^84^ NaV_2_(PO_4_)_3_,^83^^,^^85^^,^^86^ and Ca_x_ Na_0.5_VPO_4.8_F_0.7_ (Figure 4B).^87^ It is worth mentioning that Na ions are still present in most of these compounds, and their role during cycling remains to be fully assessed. K-vacant KVPO_4_F was used as a cathode in a Ca-ion cell after full electrochemical removal of potassium. Specific capacity of *ca.* 60 mAh/g was obtained in Ca half cells, yet with limited Coulombic efficiency.^88^ Significantly higher specific capacity (ca. 140 mAh/g) was achieved using NaV_1.5_Cr_0.5_(PO_4_)_3_ host structure, obtained from the electrochemical desodiation of Na_3_V_1.5_Cr_0.5_(PO_4_)_3_, benefiting from an additional valence electron of Cr.^89^ DFT-based calculations were used to screen Ca-NaSICON type compounds with the general formula Ca_x_M_2_(ZO_4_)_3_ (Z = Si, P, S and M = Ti, V, Cr, Mn, Fe, Co, and Ni) as possible Ca cathodes. Ca_x_V_2_(PO_4_)_3_, Ca_x_Mn_2_(SO_4_)_3_, and Ca_x_Fe_2_(SO_4_)_3_ were found to be promising cathode materials in terms of intercalation voltages, thermodynamic (meta)stabilities, and migration barriers.^90^ Another theoretical screening study of Ca intercalation in a wide range of chemistries has identified α-VOPO4 as a promising cathode material.^91^ Yet, the feasibility of the synthesis and experimental electrochemical performances of many computationally predicted Ca hosts remains to be demonstrated.

Overall, several different classes of inorganic materials exhibit a level of reversible electrochemical cycling with non-aqueous Ca electrolytes, and they are very rarely tested with Ca metal anode-compatible electrolytes. This represents a key gap in current cathode host reports and should be addressed in future studies. Additionally, the insertion/de-insertion of Ca^2+^ ions reversibility often remains questionable due to potential contributions of proton cycling and pseudo-reversible electrolyte degradation. Hence, much more effort should be directed at the identification of Ca^2+^ host sites through complementary characterization techniques directed at both bulk and surface characterization of active materials.

#### Conversion cathode materials

Conversion-active materials offer a possibility to circumvent limitations connected with slow Ca^2+^ cation solid-state diffusion into rigid inorganic hosts. Among them, sulfur is especially promising due to its high capacity, low cost, and abundance, which have already incited significant research efforts in the field of Li-S and other metal-S batteries. Several attempts have been made to demonstrate reversible Ca-S batteries. The first attempt was a Ca metal-S battery in Ca(ClO_4_)_2_/ACN electrolyte,^93^ which could only be used as a primary cell due to Ca passivation. The first reversible Ca-S battery was demonstrated using a mixed Li/Ca electrolyte in LiCF_3_SO_3_/Ca(CF_3_SO_3_)_2_/G4.^94^ The presence of Li^+^ ions significantly increased the utilization of sulfur and reversibility. However, it has to be noted that Li was found in discharged cathode material. Additionally, its discharge voltage was below 1.5 V, a value much lower than expected, hinting toward Ca metal passivation.

The first rechargeable Ca-S battery utilizing Ca(B(hfip)_4_)_2_/G1 exhibited significantly higher discharge voltage, close to 2.1 V (Figure 4D).^92^ Utilization of active material in a simple S/C cathode composite was somehow limited with the maximum attainable capacity of 800 mAh/g and relatively rapid capacity fade, which dropped below 200 mAh/g already after 10 cycles. A similar study employing AC cloth demonstrated a slightly higher discharge capacity of sulfur. Electrochemical testing was complemented with an extensive spectroscopic characterization, which confirmed the reduction of sulfur through the formation of polysulfides and the formation of Ca sulfide, which was found to be reversible.^95^ Later, Se cathode was investigated, as an option to avoid the issue of low electronic conductivity of S. Although Se reduction was reversible according to the spectroscopic characterization, electrochemical performance was plagued by poor utilization and rapid capacity fade.^96^ Significantly improved cyclability was achieved with a CuS-based cathode, demonstrating 500 cycles, although this was at a very high current density and with severely limited capacity.^97^ While poor cyclability of S and Se cells can be in part attributed to the inherent passivation of Ca metal anode, the effect of soluble sulfur species on Ca metal anode should be investigated to determine the future direction of Ca-S research. Future research should also explore possibilities of increasing S loading and controlling electrolyte amount to assess the practical viability of the Ca-S battery as a high-energy-density battery technology.

A very intriguing option is oxygen (O_2_) as an active cathode material with an exceptionally high capacity of 3,350 mAh/g upon theoretical conversion to oxide (O^2−^). However, the full O_2_ reduction reaction includes a four-electron multi-step reaction, as well as the generation of highly active intermediate products, raising many practical challenges. Recently, oxygen reduction to peroxide (O_2_ to O_2_^2−^) has been claimed within the framework of a Ca metal flexible battery, capable of operation in an ambient atmosphere.^63^ However, many details, including the operation of the Ca metal anode in the presence of oxygen, were not clearly presented and require further confirmation.

#### Organic cathode materials

Organic cathode materials with their adaptable structures offer a unique opportunity to overcome the limitations of rigid inorganic hosts. Organic active materials can be, according to their electrochemical mechanism, classified as n-type, p-type, and bipolar materials. However, if the targeted battery cell should only shuttle Ca^2+^ ions and enable high-energy density with a Ca metal anode under lean electrolyte conditions, the cathode active material should belong to the n-type class to enable storage of Ca^2+^ cations. The first demonstration of Ca^2+^ ion storage was done on a carbonyl-type material, perylenetetracarboxylic dianhydride (PTCDA), in aqueous media.^98^ The PTCDA compound was later studied in non-aqueous electrolytes based on carbonate solvents.^99^ The first proof-of-concept of Ca metal anode-organic cathode cell was demonstrated using poly (anthraquinonyl sulfide) (PAQS) polymer based on anthraquinone (AQ) functionality, with very good capacity utilization of 75%. Nevertheless, limited cycling stability could be achieved due to a gradual passivation of the Ca metal anode in the Ca(B(hfip)_4_)_2_/G1 electrolyte.^45^ In a 3-electrode setup, in which the Ca metal anode overpotential was not the performance-limiting factor, better cyclability was obtained. However, accelerated capacity fade compared with the Li battery cell was still observed. The electrochemical mechanism was investigated through *ex situ* infrared (IR) spectroscopy (Figure 4G) and scanning electron microscopy (SEM)-EDS, which revealed a reversible reduction of the carbonyl bond upon discharge and utilization of both Ca^2+^ cations and CaA^+^ ion pairs in the electrochemical mechanism. Utilization of CaA^+^ ion pairs can lead to a decrease of practical battery capacity due to the electrolyte salt consumption in the electrochemical reaction,^100^ as well as a potential contribution to decreased cycling stability due to decomposition of the electrolyte.^101^ PAQS was later utilized as cathode material in combination with a Ca-Sn alloy anode and the use of different cathode preparation procedures. In this cell setup, 1,000 cycles were demonstrated, but capacity utilization and retention remained limited to 45 mAh/g (20% theoretical).^102^

A similar polymer, polyanthraquinone (PAQ), was also used in combination with Ca-Sn alloy present in a large-capacity surplus and demonstrated even better cyclability up to 5,000 cycles.^5^ PTCDA and other smaller aromatic dianhydride building blocks, as well as derived polyimides, were also investigated as active materials.^103^^,^^104^ Electrochemical testing in a symmetrical cell setup displayed excellent rate capability up to 50 C and long-term cycling, and practical active material utilization was limited to around 68% for larger perylene-derived compound and 45% for more compact naphthalene-based one. Encouraging high-rate performance of organic materials in Ca cells opens up an opportunity to develop high-power Ca cells. Among p-type materials, polytriphenylamine (PTPAn) has been quite intensively investigated in different electrolytes.^105^^,^^106^ In all cases, the PTPAn showed good reversibility, demonstrating compatibility with a variety of salt anions and solvents. A breakthrough in the redox potential of n-type organic materials was achieved by utilizing amorphous coordination polymers based on the Ca-Zn-PTtSA (PTtSA [benzene-1,2,4,5-tetra-methylsulfonamide]) active group. Ca-Zn-PTtSA displayed good electrochemical reversibility and a voltage higher than 3 V in Ca metal cells.^107^

Although organic materials offer excellent reversibility and seem to be the currently best-performing Ca host material, they still do not deliver as high-capacity utilization and retention as in Li electrolytes. At the same time, we have to note that many organic electrode parameters, like active material loading, electrolyte amount, and required organic electrode porosity, still remain to be tested in more prototype-oriented cell setups to guide research toward higher TRL (technology readiness level).^23^ At the same time, a shift to higher-energy-density active materials is needed from current model compounds with relatively modest capacities and redox potentials.

### Toward practical Ca batteries

**Figure 5:**
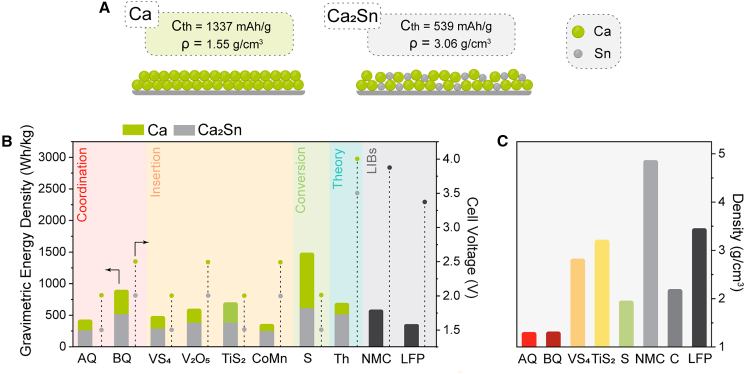
Projected energy density of Ca batteries based on different active materials (A) Comparison of Ca metal and Ca_2_Sn as anode materials. (B) Examples of coordination, insertion, and conversion of cathode materials and theoretical high-voltage cathode (4.0 V and 200 mAh/g) for comparison—voltages and energy densities of batteries employing Ca and Ca_2_Sn as negative electrodes. All energy density calculations consider only the mass of active electrode materials without considering non-active materials such as electrolyte, separator, current collectors, and casings. (C) Comparison of densities of selected materials, with NMC, graphite, and LFP as current LIB standard cells.

The Ca metal anode is the main driving force for the development of Ca batteries. However, due to the passivation of Ca metal, Sn-based compounds are also currently considered as potential anode materials.^5^^,^^102^ Although CaSn_3_ currently appears to be the active phase, it has a much lower capacity (135 mAh/g) than the more calciated Ca_2_Sn (539 mAh/g). Graphite, the standard Li-ion anode, has also been investigated and shown to reversibly intercalate Ca^2+^. However, similar to sodium, intercalation of Ca^2+^ occurs only through co-intercalation of solvent (glyme or DMAc), and only moderate reversible capacities were achieved (below 100 mAh/g),^108^^,^^109^ which significantly limits the achievable energy density. In Figure 5, various Ca battery scenarios are considered, and energy densities are calculated (considering only active materials) based on different cathode materials combined with two anodes: Ca metal and Ca_2_Sn alloy. The latter alternative anode was considered because Sn-based intermetallic compounds can typically offer the highest Ca content. Three different classes of cathodes were considered—inorganic (VS_4_, V_2_O_5_, and Ca_3_CoMnO_6_ abbreviated as CoMn), conversion (sulfur), and organics based on the AQ and benzoquinone (BQ) active groups, as well as a theoretical, not yet available, ideal high-voltage cathode exhibiting 4 V vs. Ca^2+^/Ca and a capacity of 200 mAh/g.

The use of Ca_2_Sn as an electrode, due to decreased cell voltage (0.5 V) and anode capacity, leads to a significant drop in energy density, falling behind NMC (lithium nickel manganese cobalt oxide) as a current high-energy-density LIB. While the S cathode presents a clear advantage over LIB systems, the practical energy density of these cells could be significantly compromised due to the high amount of required non-active materials during cell engineering (carbon additives, electrolyte, etc.). The energy density of Ca cells becomes very promising when high-capacity cathodes (BQ and S) are being considered. On the other hand, all systems employing Ca metal anodes, even with cathodes exhibiting quite moderate voltages in the range of 2–3 V, display competitive energy densities with respect to graphite/LFP and graphite/NMC LIBs. However, unstable Ca metal interface, limited electrolyte stability window, and insufficient cathode reversibility remain key fundamental challenges. It is important to note that the practical energy density of Ca batteries similar to LIBs will have to be achieved by careful engineering and optimization of different cell parameters, such as electrolyte amount, active material loading, porosity, current collector thickness, separator, cell housing, etc. Moreover, it is yet to be confirmed if similar electrode loadings to contemporary LIBs can be achieved with non-insertion cathodes.^110^

Another key element for realizing practical high-energy-density Ca metal batteries will be the manufacturing of Ca metal anodes. Mechanical properties of metal are essential parameters affecting its processability and possible integration into a battery assembly line. While commercial Li metal anode-based batteries are currently being produced using a metal extrusion process^111^ facilitated by the low Young modulus of Li (4.9 GPa), such a strategy could be very challenging for Ca with a much higher Young modulus (20 GPa). In addition to an increased production cost, any mechanical processing of pure Ca metal can result in poorly controlled metal surface passivation. Therefore, a promising approach following the more mature Mg battery technology would be to use foils (compatible with the roll-to-roll cell assembly procedure), whose ductility can be adjusted via alloying or heat treatment.^112^^,^^113^ Calcium’s relatively high yield strength could also turn into a technological advantage in the sense that it might not require the use of a current collector. Another strategy could consist of obtaining Ca foils through deposition methods (physical or electrochemical) onto a suitable substrate. Nevertheless, the main concern for each of these strategies will be to carefully control the surface chemistry of the metal anode to prevent detrimental metal passivation. As mentioned before, the reasonable Ca^2+^ migration energy barrier in CaO could present a unique advantage of Ca over Mg if native oxide layer growth can be limited to a few nanometers during electrode processing in a dry room environment.

The Ca-metal/electrolyte interphase could also be controlled by pretreatment with either inorganic or organic compounds. Inorganic compounds include p-group elements such as tin, bismuth, and antimony. Ca-Sn interlayers have shown improved electrochemical performance compared with untreated Ca metal and enabled reversible electrochemical operation with amide-based electrolyte. The mechanism was rationalized by a mixed layer of calcium halides and a calcium alloy, where the halide acts as an electronic insulator and the calcium alloy as an ion conductor.^114^ The surface coating of amorphous Al_2_O_3_ on a Ca surface was modeled using a combination of DFT and AIMD simulations.^115^ The calculations provided information on the energetically stable phase Ca_x_Al_2_O_3_ up to x = 1.5 and the corresponding volume change of 200% that would accompany Ca^2+^ insertion, and also confirmed that a (001)-terminated metallic Ca coated with a Ca_1.5_Al_2_O_3_ layer could prevent EC solvent decomposition. However, the stability of a realistic electrolyte was not given, and calculations of Ca diffusion coefficients in the Ca_0.2_Al_2_O_3_ phase also show extremely low values. Similar calculations on alternative materials, such as Ca halogenides already used in Ca electrolytes, could help to identify promising coatings for Ca metal foil handling in the cell assembly line.

Various plating substrates such as Au, Pt, Cu, Al, SS, and GC have been reported and shown to affect plating/stripping efficiency as well as plating/stripping overpotential.^34^^,^^35^^,^^37^ However, a systematic investigation to select the best substrate and substrate pretreatments (control of surface morphology and chemistry) is still lacking. Regarding the impact of cycling conditions, a study conducted in Ca(BH_4_)_2_/THF electrolyte has shown a clear effect of current density on the Ca deposit morphology. Transitions from planar geometry to globular and finally dendritic Ca upon increasing current densities were observed. However, dendrites began to form only at very high current density (ca. 50 mA/cm^2^),^116^ which is well above the typical critical current density at which Li dendrites usually start to grow (>1 mA/cm^2^). This suggests potential for the development of safe metal anode batteries and warrants further investigation/confirmation.

## Conclusions

Ca battery research has made significant progress in the last decade. First, by the realization of reversible Ca metal plating/stripping at elevated temperature, which was followed by the realization of Ca plating/stripping at RT and the development of WCA-based Ca electrolytes. Development in the field of electrolytes enabled investigation of new cathode materials beyond conventional insertion cathodes, where both organic and sulfur cathodes have demonstrated reversible electrochemical activity, opening avenues for future explorations of alternative Ca hosts. Nevertheless, the capacity utilization and retention still need significant improvement to close the performance gap with respect to the alkali batteries.

Although Ca electrolytes can match the standard Li-ion electrolytes in terms of ionic conductivity, we are still far from plating/stripping efficiencies of over 99.9% required for practical metal anode applications. State-of-the-art WCA-based Ca salts in glyme-based electrolytes present an unstable metal/electrolyte interphase and suffer from limited anodic stability due to the utilization of glyme-based solvents. Certain studies reported improvement in the performance through the addition of co-salts and solvation sheath additives. However, a better understanding of the Ca solvation shell and the formation of the metal/electrolyte interphase is needed to enable the design of next-generation Ca electrolytes. A silver bullet solution would be the development of an interphase layer with good Ca^2+^ transport properties and an electronically insulating nature, like the SEI in LIBs technology. Since the interphase formation mostly results from the reduction of the cation solvation shell, controlling the solvation structure in electrolyte solution appears to be the most straightforward and simple tool to engineer Ca metal interphase. An “SEI”-like metal interphase could also open up the possibility for the use of solvents that are currently incompatible with Ca metal. New solvents with higher oxidation stability would, at the same time, address the limited oxidative stability of current Ca electrolytes based on glymes. On the cathode side, inorganic cathodes have shown a certain degree of reversibility for Ca insertion that needs to be further explored and improved. Currently, the most reversible cathodes are organic and conversion types, such as sulfur, both complying perfectly with one of the most important requirements for post-Li batteries: sustainability.

Nevertheless, the development of Ca metal batteries can seem erratic due to issues connected with limited reproducibility and discrepancies between results reported in the literature. This is in part caused by the specifics of Ca metal electrochemistry, high sensitivity to impurities, and direct import of certain approaches from the field of Li-batteries. In particular, Ca/Ca symmetric cell cycling should never be considered as a standalone demonstration for Ca reversible plating/stripping, especially when exploring new classes of electrolytes. Overall, Ca battery research is still at a very low TRL, and much more work is needed to achieve performance benchmarks comparable to those of LIBs or even alternative technologies, such as Na-ion batteries, that will complement LIBs in the coming years. In particular, we believe that the general use of reliable and standardized testing protocols within the scientific community constitutes an essential aspect for the development of this emerging battery chemistry.

## Acknowledgments

O.L. and J.B. acknowledge the financial support of the Ministry of Higher Education, Science, and Innovation through the COBRA M-ERA.NET project (M-ERA.NET call 2023, C3360-25-452015); the Slovenian Research and Innovation Agency (ARIS) through research program P2-0423; and projects N2-0279, J2-4462, and J2-70086. A.P., D.S.T., and T.P. gratefully acknowledge funding from the European Research Council (ERC) under the European Union’s Horizon 2020 Research and Innovation Programme (grant agreement no. 101089281) and the Spanish Agencia Estatal de Investigación Severo Ochoa Programme for Centres of Excellence in R&D (CEX2023-001263-S).

## Declaration of interests

The authors declare no competing interests.
